# ChIP-exo interrogation of Crp, DNA, and RNAP holoenzyme interactions

**DOI:** 10.1371/journal.pone.0197272

**Published:** 2018-05-17

**Authors:** Haythem Latif, Stephen Federowicz, Ali Ebrahim, Janna Tarasova, Richard Szubin, Jose Utrilla, Karsten Zengler, Bernhard O. Palsson

**Affiliations:** 1 Bioengineering Department, University of California San Diego, La Jolla, California, United States of America; 2 Novo Nordisk Foundation Center for Biosustainability, Technical University of Denmark, Lyngby, Denmark; Niels Bohr Institute, DENMARK

## Abstract

Numerous *in vitro* studies have yielded a refined picture of the structural and molecular associations between Cyclic-AMP receptor protein (Crp), the DNA motif, and RNA polymerase (RNAP) holoenzyme. In this study, high-resolution ChIP-exonuclease (ChIP-exo) was applied to study Crp binding *in vivo* and at genome-scale. Surprisingly, Crp was found to provide little to no protection of the DNA motif under activating conditions. Instead, Crp demonstrated binding patterns that closely resembled those generated by σ^70^. The binding patterns of both Crp and σ^70^ are indicative of RNAP holoenzyme DNA footprinting profiles associated with stages during transcription initiation that occur post-recruitment. This is marked by a pronounced advancement of the template strand footprint profile to the +20 position relative to the transcription start site and a multimodal distribution on the nontemplate strand. This trend was also observed in the familial transcription factor, Fnr, but full protection of the motif was seen in the repressor ArcA. Given the time-scale of ChIP studies and that the rate-limiting step in transcription initiation is typically post recruitment, we propose a hypothesis where Crp is absent from the DNA motif but remains associated with RNAP holoenzyme post-recruitment during transcription initiation. The release of Crp from the DNA motif may be a result of energetic changes that occur as RNAP holoenzyme traverses the various stable intermediates towards elongation complex formation.

## Introduction

Crp (cAMP receptor protein; also known as CAP, catabolite activator protein) is the most thoroughly characterized transcription factor from a structural and mechanistic standpoint [1–3]. It has been the subject of numerous studies focused on unraveling the drivers behind transcription factor activation. These have included, to name a few, comparisons of nuclease protected DNA fragments to elucidate the Crp consensus motif sequence [4–7], mutational analysis of Crp and/or RNA polymerase (RNAP) to reveal the binding interactions that form for distinct promoter architectures [8–15], and three-dimensional structures of Crp and models of it in complex with DNA and RNAP that have been formed [2,16–19]. However, the analysis of Crp and other transcription factors is limited to the *in vitro* model systems for which they are confined and have largely focused on the steps leading to recruitment of RNAP holoenzyme with little attention on the subsequent stages of initiation.

DNA footprinting studies have been instrumental to our understanding of promoter mechanics. This classic approach utilizes the protection from nuclease digestion provided by proteins bound to DNA to produce a highly precise map of the binding site [20]. This method has been extensively applied to study the mechanics and kinetics of transcription initiation events [21–23]. The outcome of these studies and complementary characterization studies (e.g., x-ray crystallography, single-molecule approaches, and predictive modeling) are at the core of our current, multi-step model of transcription initiation [21–24]. However, the rate at which RNAP proceeds through transcription initiation is typically too rapid to be differentiated under physiologically relevant conditions. For example, numerous temperature-modulating experiments have shown that the open RNAP complex dominates at physiological temperatures and that reduced temperatures are needed to recover closed complex intermediates [25–28].

DNA footprinting has also played a significant role in our current understanding of transcription activation by Crp. Detailed *in vitro* studies performed on model promoters (e.g., *lac*, *galP1*, and *deoP2*) have yielded three classes of Crp promoters depending upon the location of the consensus motif sequence(s) relative to the transcription start site (TSS), the number of motif sequences, and the presence of additional transcription factors [1,2]. Class I promoters are thought to mediate activation through a simple recruitment mechanism where interactions are formed between Crp and the α subunit of RNAP yielding the closed promoter complex. Crp forms up to three interactions with RNAP holoenzyme and facilitates isomerization to the open promoter complex at Class II promoters. Class III promoters involve two Crp molecules and a second transcription factor that often represses the activating action of Crp. Footprinting studies under highly controlled and stabilizing conditions have shown that the Crp motif sequence is protected when in complex with Crp and RNAP holoenzyme [29–32]. However, these interactions were studied in stabilizing *in vitro* conditions with a focus on characterizing early events during transcription initiation.

Chromatin immunoprecipitation (ChIP) followed by microarray hybridization (chip) or next-generation sequencing have provided genome-scale information on DNA/protein interactions *in vivo*. These techniques have been paramount to studying transcriptional regulators and to construct regulons and transcriptional regulatory networks. However, the information ascertained by application of these methods predominantly provides a binary (present/absent) representation of binding events. Integrating with gene expression analysis allows for expansion of these binary calls to provide conditional activation/repression calls. However, the resolution of ChIP-chip (on the order of kilobases) and ChIP-seq (on the order of hundreds of base pairs) does not enable research to precisely determine the location of the binding event. One of the challenges facing biology is to be able to predict promoter activity. One potential approach to achieve this is by obtaining high-resolution mechanistic information of individual promoters and to convert that mechanistic information into a model of promoter dynamics.

An enhanced form of ChIP-seq called ChIP-exonuclease (ChIP-exo) [33] generates genome-scale maps of DNA binding proteins at single nucleotide resolution. This enables precise identification of binding events by combining DNA footprinting with ChIP. For instance, this method has been applied to the study of eukaryotic pre-initiation complexes, which is typical comprised of RNAP II and no less than six additional general transcription factors [34]. The ChIP-exo results were able to spatially resolve individual proteins and agreed strongly with findings produced from crystallographic models. We have previously applied this footprinting assay for application in *Escherichia coli* to elucidate the Fur transcriptional regulon, which predominantly is found to act as a repressor [35].

The study of bacterial transcription activation using high-resolution ChIP-exo data could affirm the transcription initiation processes elucidated *in vitro* under *in vivo* conditions and extend those observations to the genome-scale. Crp provides an ideal entry point for such a study because of the mechanistic and structural information borne out through decades of detailed work on individual promoters [1,2,36–39]. Here, we applied ChIP-exo to study the DNA protection patterns generated by the housekeeping sigma factor, σ^70^, with respect to published data on RNAP holoenzyme footprinting data. We then compared the protection pattern provided by Crp to σ^70^ and surprisingly found tremendous overlap in their DNA footprinting pattern. However, there was very little observed protection of the Crp motif sequence. This phenomenon was then explored in a repressor, ArcA, and the Crp familial protein, Fnr. Lastly, genetic perturbations to Crp/RNAP interactions were introduced and the affects of these mutations were characterized using ChIP-exo.

## Results

### Strand oriented peak distributions reveal stable intermediates in transcription initiation

**Fig 1:**
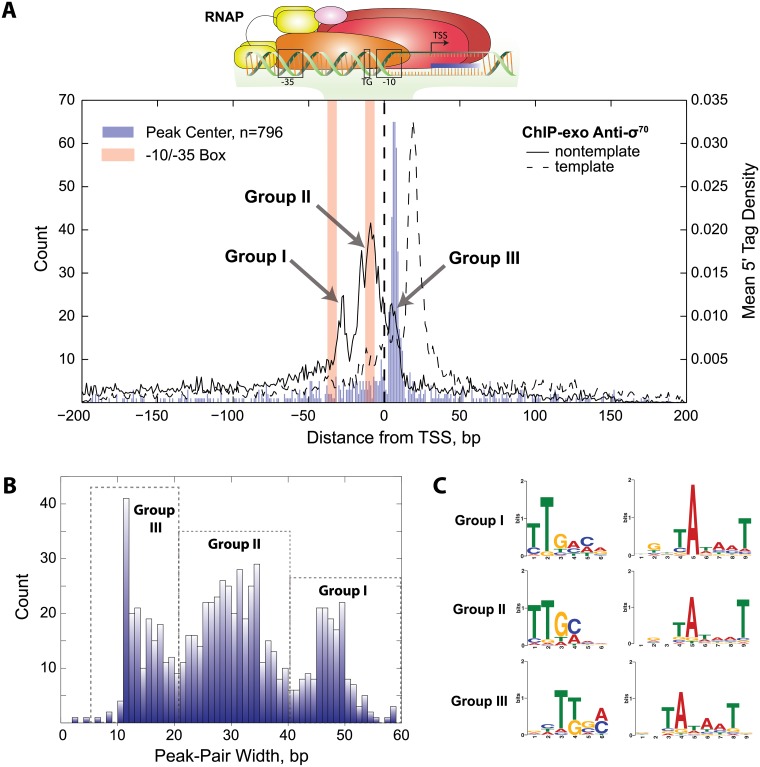
TSS aligned and oriented σ^70^ ChIP-exo peaks reveals DNA footprint patterns consistent with stable transcription initiation intermediates. (A) ChIP-exo peak regions aligned and oriented relative to the TSS. The peak center (blue bars) is shown to be downstream of the TSS with a median of 5 bp. The mean distribution of the 5’ tags is shown for both strands. The template strand distribution shows a unimodal profile that spans +20±7 bp indicative of RPO, ITC, and TEC stable intermediates. The nontemplate strand shows a multimodal distribution with modes centered approximately +5 relative to the TSS (Group III), upstream and over the -10 promoter element (Group II), and slightly downstream of the -35 promoter element (Group I). (B) Examination of the distance between template and nontemplate strand peak maximum shows that the footprint lengths are >40 bp, 21 to 40, <20 and for Group I, Group II, and Group III respectively. (C) A motif search was performed for the -10 and -35 promoter elements for Group I, Group II, and Group III promoters. All three show σ^70^-like promoter sequences with slight differences. Group I has a -35 motif that most closely resembles the consensus (TTGACA), has a highly conserved -11A, and a partial TGn motif. Group III has the least conserved -35 promoter element and no extended -10 promoter element.

The σ^70^ ChIP-exo peak distribution provides the bounds of protected DNA regions on the template and nontemplate strand. ChIP-exo profiles across all binding sites were calculated for both the template and nontemplate strand by first calculating the density of the 5’ end of tags for each individual peak region spanning 400 bp centered and oriented relative to the TSS (transcription start site). The median position of the σ^70^ peak center is 5 bp downstream of the TSS therefore the peak center is found to be an accurate approximation for the TSS (see S1 Text for detailed discussion). Furthermore, the ChIP-exo profiles for σ^70^ reveal distinctions between the template strand and the non-template strand (Fig 1A and S1 Fig). The binding profiles show a unimodal distribution on the template strand, whereas a multimodal distribution is seen on the non-template strand. The width of the peak regions was determined by calculating the distance between the maxima on the template and nontemplate strands (Fig 1B). This indicates that most promoters have a σ^70^ ChIP-exo profile that predominantly fall into one of three groupings.

The activity of lambda exonuclease is 5’ to 3’ [40] and, as such, the protected region on the template strand is found downstream of the TSS. The unimodal ChIP-exo distribution on the template strand has a maximum 5’ tag density +20 bp downstream of the TSS and approximately 30% of the mean 5’ tag density is found between 20±7 bp. The position of the unimodal distribution on the template strand is in strong agreement with numerous *in vitro* footprinting studies in model promoter constructs characterizing the stable intermediates leading to open complex (RP_O_) formation, the RP_O_, the initial transcribing complex (ITC) and the transition to the ternary elongation complex (TEC). However, the closed promoter complex (RP_C_) does not have an advanced footprint extending to the +20 position.

Unlike the template strand, the ChIP-exo 5’ tag distribution for the nontemplate strand is multimodal. This distribution marks the upstream boundary relative to the TSS. The dominant mode found between -18 and -1 accounts for 28% of the 5’ tag density. Therefore, promoters that belong to this mode have partial to complete protection of the discriminator sequence, the -10 promoter element, and the TGn extended -10 element but little to no protection of the -35 promoter element or any upstream promoter elements (e.g., UP element). The -35 promoter element is partially protected by the mode farthest upstream which accounts for 9% of the 5’ tag density profile and spans -34 to -23 with a maximum located at -28. The upstream boundary, -3, is located in the center of the -35 element. The downstream mode accounts for 8% of the 5’ tag density and is located downstream of the TSS. The boundaries of this mode are between +4 and +12 with a local maximum at +6. Like the template strand, the DNA protected regions of the different modes on the nontemplate strand provide little to no support that recruitment and RP_C_ complex formation is being captured by ChIP.

### Discussion of σ^70^ ChIP-exo data in the context of *in vitro* transcription initiation studies

Collectively, the ChIP-exo distribution of the mean 5’ tag density on both strands indicates that σ^70^ is being capturing in complex with RNAP when assessed in the context of *in vitro* footprinting studies performed on model promoter constructs. Furthermore, both the template and nontemplate strands provide evidence that σ^70^ ChIP studies identify stable intermediates during transcription initiation that occur after the recruitment of RNAP and RP_C_ formation. The template strand protected regions in the σ^70^ ChIP-exo profiles provide the strongest evidence that post-recruitment stable intermediates are being captured. The template strand distribution protection extends to +20 relative to the TSS, which agrees with numerous *in vitro* studies that have demonstrated a clear transition in the downstream protected boundary from -5 to +5 in RP_C_ to +20 for RP_O_, ITC, and early TEC complexes (9–12). Hydroxyl radical footprinting studies on RP_C_ formation in the T7A1 promoter showed that the short-lived RP_C_ complex protects DNA to approximately -5 bp (13–15). Similar results were observed in DNase footprinting of the T7A3 promoter, *lac*UV5, and *rrnB*P1 (16, 17). Furthermore, an RNAP mutant with deficient open complex formation was found to have DNase footprints that extend to just +1 at the λP_R_ promoter (18). However, the RP_C_ complex was only observed when the temperature was dropped in most of these studies. The temperature dependent capture of early closed complexes has been shown to be a result greater RP_O_ abundance at physiological temperatures (14, 15, 17, 19). Conversely, the advanced downstream boundaries centered on +20 has been observed in studies performed on the intermediates leading to RP_O_ and the RP_O_ complex for the T7A1 promoter (13–15), the T7A3 promoter (17), the *rrnB*P1 promoter (16, 20–22), the λP_R_ promoter (19, 23), and the *lac*UV5 promoter (17, 24). Furthermore, the ITC and the transition to the TEC also have a downstream footprint boundary of +20–25. DNase footprinting of T7A1, *tac*, and *lac*UV5 promoters showed that the ITC has a slightly advanced footprint at +25 compared with +20 for RP_O_ and the early TEC had a footprint at +30 (25–27).

ChIP-exo mean 5’ tag density profiles for σ^70^ on the nontemplate strand show a multimodal distribution with regions that provide protection to different components of σ^70^ promoter elements. These were found to form three modes spanning -34 to -23, -18 to -1, and +4 to +12 with the region spanning -18 to -1 accounting for the largest fraction of the 5’ tag density profile. While periodic patterns of DNA protected regions at the upstream boundary are common (9, 11, 12), the location of the boundaries is supportive of post-recruitment intermediates (27–29). A detailed study on the *lac*UV5 promoter using DNase I, methylation protection, and exonuclease III protection across transcription intermediates showed that transitions from RP_O_ to ITC undergoing abortive initiation retained strong protection of a region between -24 and -6 to exonuclease III digestion that was reduced to protection of the region downstream of -6 after escaping the abortive transcription phase to produce longer transcripts (27). This is further corroborated by a recent study that showed the *lac*UV5 promoter has an upstream footprint boundary at -23 in the presence of σ^70^ compared with -13/-14 for the σ^70^ lacking transcribing complex (29). A study of the T7A1 promoter using exonuclease III showed a drastic movement in the upstream-protected region from -43 to -3 in the transition from RP_O_ to early transcribing complexes (ITC or TEC) (28). Furthermore, the width of the protected region agrees with studies examining RPO, ITC, and TEC. Early TECs have been found to have footprint regions spanning ~30 bp whereas RP_O_ and ITC have longer footprint seen to be 50+ bp in length (25, 27, 30).

Kinetic studies also support the notion that the ChIP-exo data presented here is reflective of stable intermediates occurring post-recruitment of RNAP and RP_C_ formation. Genome-scale characterization studies of bacterial transcription have shown the rate-limiting step in transcription predominantly occurs post-recruitment of RNAP (7, 31, 32). For example, the λP_R_ promoter is limited at the opening of the transcription bubble marked by a slow transition from the closed intermediate to the open intermediate (19, 23, 33–35). Furthermore, the promoter λP_R’_ encodes a promoter-proximal pause site induced by a -10 like sequence downstream of the TSS (36). A similar pause occurs in the *lac* promoter (37, 38) and, in fact, it is estimated that the occurrence of promoter-proximal pausing is upwards of 20% in *E*. *coli* (38–40). However, numerous additional processes along the trajectory of RP_O_ to TEC formation have been found to be rate determining including scrunching (41), and promoter escape (10, 32). Therefore, ascertaining the potential bottlenecks in transcription initiation at genome-scale and under *in vivo* conditions would be of value to genome-scale models of promoter kinetics.

### Promoter motif analysis of the σ^70^ peak distributions

It is known that promoter sequence elements involved with RNAP holoenzyme recruitment contribute to the post-recruitment kinetics of transcription initiation [22–24]. Thus we examined the -10 and -35 promoter elements for the different σ^70^ groups (Fig 1C) as determined by the difference in peak-pairs (Fig 1B). σ^70^-like promoter motifs were found in all three groups. Group I, having the longest distance between peak-pairs, has a motif that most resembles the -35 consensus sequence (TTGACA). Furthermore, the -10 promoter element has near perfect consensus at the critical -11A position and a partial TGn motif characteristic of the extended -10 promoter element. Group II resembles the motifs found in Group I but with lower sequence conservation in both the -10 and -35 promoter elements. Conversely, Group III has the most divergent -35 motif from consensus and no appreciable motif for the extended -10 promoter element.

### Promoter characterization of the canonical transcriptional activator, Crp

**Fig 2:**
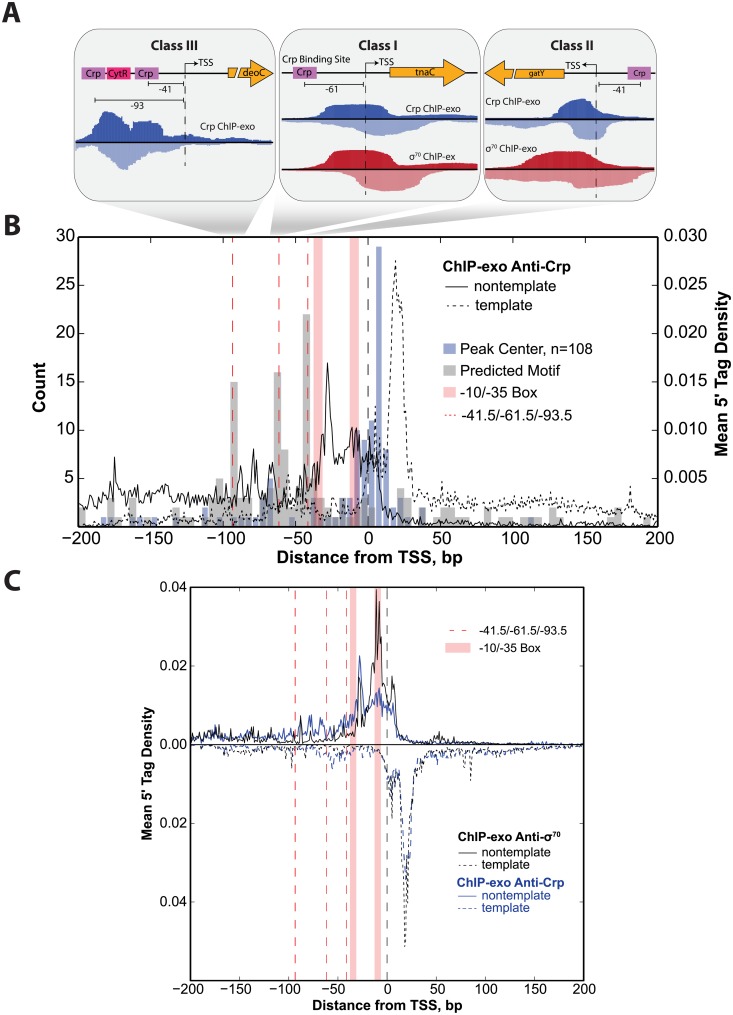
Crp and σ^70^ have highly similar ChIP-exo footprints. (A) Gene tracks are shown that exemplify the different Crp ChIP-exo footprint profiles observed for the three different classes of Crp promoters. At the Class III promoter *deoC* footprints are found over the Crp motif and the CytR motif which sequesters Crp preventing activation. However, under the activating Class I and Class II promoters there are few observed reads over the Crp motif. Instead, the peak is centered on the TSS and the footprint region cooccurs with that found for σ^70^. Examples of this are shown for *tnaC* (Class I) and *adhE* (Class II). (B) Shown is the mean 5’ tag density ChIP-exo profile aligned and oriented relative to the TSS generated for Crp grown on glycerol minimal media. The distribution of the center position at +23 across all Crp peak regions (blue bars) shows close proximity to the TSS. The template strand distribution (dashed black trace) corresponds with the downstream region centered at +20 that is associated with stable intermediates of the RPO, the ITC, and the TEC as was observed for σ^70^. The nontemplate strand distribution indicates protection of DNA predominantly occurs downstream of the -35 element with little protection at the predicted binding sites (gray bars). (C) An overlay of the mean 5’ tag density profile of all Crp peak regions (blue traces) and the associated σ^70^ mean 5’ tag density profile in those same peak regions (black traces) illustrates the strong co-occurrence of Crp footprint regions with σ^70^.

Transcription factor binding was further studied with ChIP-exo in *E*. *coli* Crp. ChIP-exo data showed strong consistency with previously determined Crp binding sites (see S1 Text). ChIP-exo profiles enabled high-resolution distinction of DNA protection patterns among the three classes of Crp promoters, which are briefly reviewed in S1 Text. Representative examples of ChIP-exo profiles generated for cultures exponentially growing in glycerol minimal media (a Crp activating condition) are shown for each of the three Crp Classes (Fig 2A). The *deoC* promoter is a Class III promoter with two Crp binding sites flanking a CytR regulatory site that represses the activating action of Crp [41]. The ChIP-exo protected regions are in close proximity with the three consensus motif sequences with protected regions near -40 and -90 as previously seen *in vitro* [41]. However, markedly different profiles are observed in the Class I (*tnaC*) and Class II (*gatY*) promoters that often have no exonuclease protection to the Crp binding site, but instead, have strong protection of the region surrounding the TSS. In fact, these regions correspond greatly with the ChIP-exo profiles generated for σ^70^ under the same condition but no observed σ^70^ ChIP-exo peak was detected for the repressed *deoC* promoter.

The results for these individual promoters are consistent when extended to the genome-scale. Analogous to the analysis performed on σ^70^, all Crp ChIP-exo binding profiles were aligned and strand-oriented relative to the TSS. The same was done with the peak center position and the predicted Crp motif sequence (Fig 2B). Examination of the motif sites shows three regions of elevated Crp motif sequences centered at -41.5, -61.5, and -93.5 bp upstream of the TSS corresponding with the expected positions of Class II, Class I and Class III promoters respectively [1,2]. However, the mean 5’ tag distribution of Crp ChIP-exo data oriented relative to the TSS illustrates that the peak centers align greatly with the TSS and not the Crp binding site. A similar ChIP-exo profile was obtained when wild type *E*. *coli* was grown on fructose, another Crp activating condition, but when grown on glucose, a Crp repressing condition, few binding sites were detected and poor alignment was observed relative to the TSS (S2 Fig). We further verified that these results were not artifacts attributed to the anti-Crp antibody used to perform ChIP-exo by generating data on a Δ*crp* strain and no correlation was observed between biological replicate datasets indicating minimal impact due to non-specific binding (S3 Fig). Therefore, the Crp binding profile under activating conditions has poor alignment with the consensus motif sequence.

The ChIP-exo 5’ tag density profile for Crp was also compared with σ^70^ across all Crp binding regions (Fig 2C). Strand orientated Crp density profiles reveal a unimodal distribution on the template strand and a multimodal distribution on the nontemplate strand analogous to those found for σ^70^. The template strand strongly overlaps the one observed for σ^70^ with a downstream boundary of protected DNA centered on +20 accounting for 33% of the aggregate density profile. However, the Crp nontemplate density profile has distinctive features. First, there is increased DNA protection on the nontemplate strand between the -93.5 and -61.5 markers. This region encompasses 13% of the total 5’ tag density profile. These positions signify the center position of many Class III and Class I Crp motif sequences respectively [1,2]. However, none of these regions indicates protection of the Crp motif sequences found for Class I and Class III promoters and only partial protection for Class II promoters due to the overlap with the -35 box. The strong overlap with the σ^70^ binding profile and alignment with the TSS suggests that Crp immunoprecipitation is occurring in complex with RNAP holoenzyme and, as such, the ChIP profile is more reflective of the stable RNAP intermediates discussed above.

### Rifampicin treated Crp ChIP-exo

Rifampicin (rif) prevents transcription elongation beyond a length of 2–3 nt [42] and, in doing so, leaves the transcription machinery unable to advance beyond the ITC. Therefore, ChIP-exo was performed on cultures treated with rif prior to harvest followed by immunoprecipitation of Crp. The resulting mean 5’ tag density profile generated on both the template and nontemplate strand closely resembles that obtained in the non-rif treated sample (S4 Fig). Therefore, this chemical perturbation of the transcriptional state had no impact on the Crp ChIP-exo distribution and no additional upstream protection of the Crp binding site was observed. This result indicates that the exonuclease footprints are occurring on initiation complexes occurring prior to the TEC. This observation coupled with the evidence against the short-lived RP_C_ complex strongly suggests that the Crp promoters studied here are being captured after dissociation from the motif while they are still bound to RNAP. The capture seems to occur at stable intermediates formed between RP_O_ and the ITC but prior to promoter escape.

### Distinct ChIP-exo profiles for transcriptional activators and repressors

**Fig 3:**
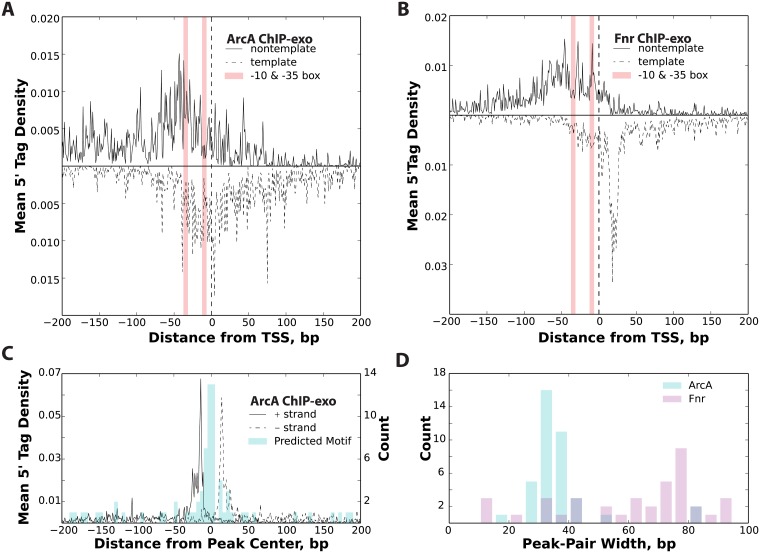
Contrasting ChIP-exo profiles of repressors and activators. (A) The TSS aligned ChIP-exo profile for ArcA, a predominantly repressive transcription factor, is shown to lack the characteristic distribution of mean 5’ tag density observed on both the template and nontemplate strand. (B) The TSS aligned mean 5’ tag density profile for Fnr, typically an activator, resembles the profile found for Crp and σ^70^. (C) The ArcA ChIP-exo profile is shown for all peak regions aligned to the peak center position. Also shown is a histogram of the center of the predicted ArcA binding site relative to the peak center position. This illustrates that the ChIP-exo profile is centered on the predicted binding site. (D) A comparison of the peak-pair distance is shown to illustrate the difference in resolution observed between ArcA and Fnr. ArcA, the repressor, is revealed to have shorter footprints compared with Fnr, the activator.

The ChIP-exo binding profiles of activating transcription factors are very different than ChIP-exo profiles of repressing transcription factors. Previous studies have shown transcription factor binding profiles centered on the regulatory motifs in eukaryotic systems [33,34,43]. Furthermore, we have seen motif centering when ChIP-exo was applied to characterizing the transcriptional repressor Fur in *E*. *coli* [35]. Therefore, we sought to examine if the alignment to the TSS seen in Crp could be extended to the familial protein Fnr and contrasted with the profile generated for a predominantly repressing transcription factor ArcA. ChIP-exo was performed on c-Myc tagged strains of ArcA (repressor) and Fnr (Crp family activator) grown anaerobically on glucose minimal media. The data generated was then processed, aligned, and oriented relative to the nearest TSS (Fig 3). ArcA, which typically occludes the TSS [44], has no defined ChIP-exo 5’ tag distribution on either strand though there is a noticeable increase in the 5’ tag density around the TSS (Fig 3A). In contrast, Fnr demonstrates a similar 5’ tag density profile as was seen for Crp and σ^70^ with a strong unimodal distribution on the template strand at +20 and a less defined modal distribution on the nontemplate strand (Fig 3B). The ArcA ChIP peak regions were aligned relative to the peak center position (Fig 3C). This resulted in a uniform distribution of 5’ tag density with sharp peaks on the forward (+) strand and the reverse strand (-). Furthermore, plotting the predicted binding sites shows that the protected regions are centered on the ArcA motif. Lastly, the peak-pair differences for ChIP-exo profiles of ArcA and Fnr are shown (Fig 3D). This reveals that the footprint obtained for the repressor is approximately 30 bp and centered on the binding motif while Crp family activators have a broader footprint distribution centered on the TSS and with a template strand footprint advanced to the +20 position. The broader footprint and advancement to the +20 position affirms the presence of RNAP holoenzyme in the immunoprecipitated complex with little to no protection of the activating motif sequence.

### Genetic perturbation of RNAP holoenzyme/Crp interactions

**Fig 4:**
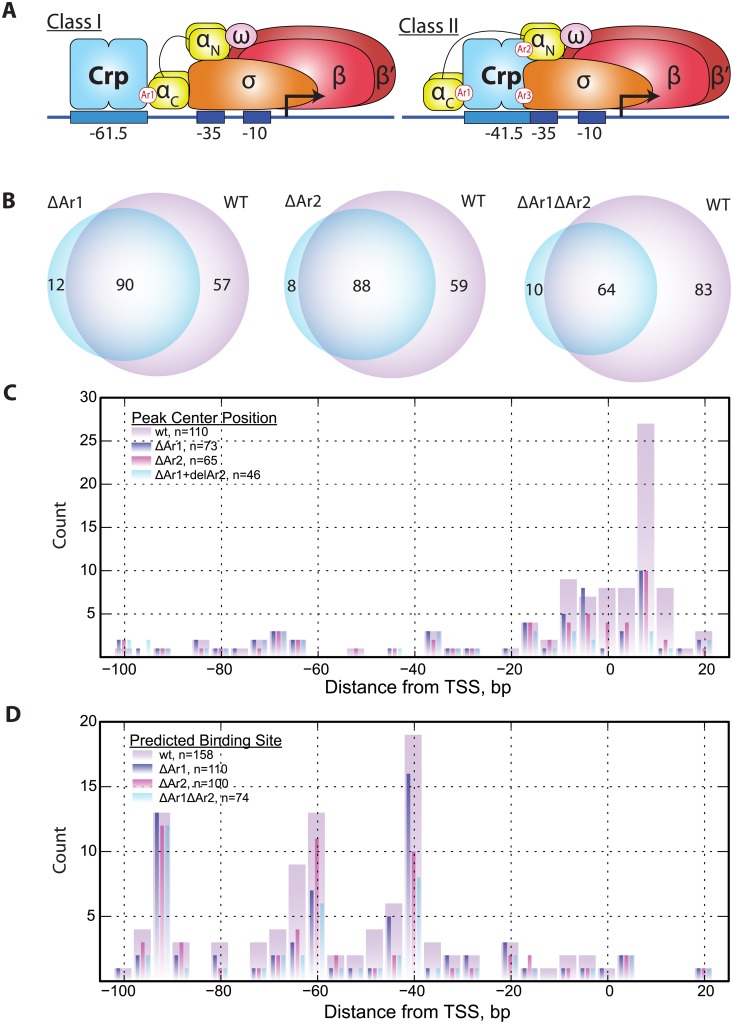
The effect of genetic perturbation on Crp/RNAP interactions. (A) Cartoon illustrating the interactions between activating regions (Ar’s) and RNAP for Class I and Class II activators. Crp Class I promoters make a single contact with RNAP at Ar1 whereas Crp Class II activators make upwards of three contacts (Ar1, Ar2, and Ar3). Deletions of Ar1, Ar2, and Ar1+Ar2 where generated. (B) Venn diagram showing pairwise comparison of peaks regions detected for ΔAr1, ΔAr2, and ΔAr1ΔAr2 with wild type Crp. All cultures were grown with glycerol as the carbon source. The mutations to Crp result in fewer detected peaks relative to wild type Crp indicating promoter destabilization. (C) Histogram of the peak center position relative to the TSS for wild type Crp, ΔAr1, ΔAr2, and ΔAr1ΔAr2 mutants. This illustrates that the peak centers nearest the TSS (-15 to +20) are predominantly affected by deletion of Ar1 and Ar2, whereas peak regions centered upstream of the TSS (< -15) are largely unaffected. (D) An alternative view to the histogram shown in (C) that shows the distribution of predicted Crp binding sites relative to the TSS. The ΔAr1 strain shows a reduction in the number of peak regions with -61.5 motifs compared with wild type and ΔAr2 indicating a sensitivity of Class I promoters to mutations to this region. Similarly, the ΔAr2 strain shows a substantial loss of Class II associated peak regions (-41.5 binding sites) compared with Class I (-61.5). The ΔAr1ΔAr2 mutant shows reductions in both -41.5 and -61.5 binding sites compared with the wild type. None of the ΔAr strains showed a reduction in the peak regions with Class III binding sites (e.g., -93.5 binding sites).

We next sought to determine the impact of genetic perturbations to the RNAP holoenzyme/Crp interactions by introducing deleterious mutations to the Activating Regions (see S1 Text for discussion) Ar1 and Ar2. Mutations were introduced to create ΔAr1, ΔAr2, and ΔAr1ΔAr2 mutants (Fig 4A). ChIP-exo was performed on these mutant strains with glycerol as the sole carbon source. In comparison with the wild type, each mutant resulted in the loss of peak regions (Fig 4B). The most drastic effect was observed in the ΔAr1ΔAr2 mutant which retained ~40% of the peaks in the wild type strain. This result indicates the importance of these Ar interactions on the stabilization of both Crp and RNAP holoenzyme at the promoter site. Furthermore, the characteristic ChIP-exo 5’ tag density profiles (see Fig 2C) on both strands were systematically degraded with each mutation resulting in profiles that no longer aligned well to the TSS (S5 Fig). To determine which peak regions were lost as a result of these genetic perturbations, the distribution of peak region centers was analyzed (Fig 4C). The mutations predominantly result in a loss of peak-regions where the peak center was located near the TSS (-10 to +20 bp) and peak centers farther away from the TSS were less impacted. Lastly, the distribution of predicted binding sites were examined in the context of the different mutant strains (Fig 4D). In agreement with expectation, modulation of Ar1 results in a drop in the predicted binding sites observed near -61.5, the typical Class I promoter distance from the TSS. This drop near -61.5 was partially recovered in the Ar2 mutant but a severe drop in the -41.5 centered binding sites occurred. This distance upstream of the TSS is associated with Class II promoters. The ΔAr1ΔAr2 mutant has a loss in peak regions with Crp binding sites matching those of Class I and Class II promoters. However, the peak regions of Class III found near the -93.5 position are unaffected by mutations in Ar1, Ar2, or both.

## Discussion

Here we present high-resolution ChIP-exo datasets that enable *in vivo* characterization of transcription initiation events at the genome-scale. The detailed footprinting performed on *E*. *coli* σ^70^ are foundational to subsequent analysis of transcription activation associated with Crp family proteins. The σ^70^ ChIP-exo profiles reflect findings determined in a number of *in vitro* footprinting studies performed on individual model promoters (for detailed discussion see S1 Text). Those studies have revealed that shortly after recruitment, the RNAP holoenzyme complex advances to the +20 position relative to the TSS. The upstream footprint boundary is less pronounced following an oscillatory pattern covering different promoter elements. In strong agreement with what *in vitro* studies have revealed, the σ^70^ ChIP-exo data presented in Fig 1 shows the template strand DNA boundary located at the +20 advanced position. Similarly, the nontemplate strand data shows a multimodal distribution that poorly protects the -35 and promoter elements upstream thereof. Thus, comparison of the σ^70^ ChIP-exo data to *in vitro* footprinting profiles of RNAP holoenzyme indicate that the ChIP results generated here, and likely elsewhere, are recovering the entire RNAP holoenzyme complex. While this may not be a surprising result, the comparison also determines that RNAP holoenzyme complex is most often captured after recruitment to the promoter. The +20 advancement on the template strand is characteristic of stable RNAP holozenyme initiation intermediates that occur post-recruitment. The advanced +20 position has been observed for stable RP_C_ intermediates, RP_O_, ITC, and early TEC complexes but not for the recruited RNAP holoenzyme complex whose footprint does not extend far beyond the TSS. Given the time scale of ChIP crosslinking is on the order of minutes, it is likely that ChIP studies characterize RNAP holoenzyme at kinetically long-lived, stable states formed on the path towards a promoter-escaped, elongation complex. *In vitro* kinetic studies support the ChIP-exo data presented here where the rate-limiting step during transcription initiation is most often downstream of recruitment. Genome-scale characterization studies of bacterial promoters have also determined that the rate-limiting step in transcription predominantly occurs post-recruitment of RNAP [45–47]. Therefor, the σ^70^ ChIP-exo results affirm *in vitro* results observed in model promoter systems and extend those findings to the genome-scale and under *in vivo* conditions.

Surprisingly, the observations made for σ^70^ were also observed in datasets where anti-Crp antibodies were used to study the binding patterns of this well characterized transcription factor. Crp binding profiles did not align to the motif sequence as would be expected but, rather, were centered on the TSS. These binding profiles, like σ^70^, largely exhibit advancement of the DNA protected boundary to the +20 position on the template strand. Furthermore, the binding pattern on the nontemplate strand shows little to no protection of the well-characterized Crp binding motifs. These results indicate that Crp and RNAP holoenzyme are not only co-immunoprecipitated during ChIP experiments, but also that the subsequent Crp ChIP-exo footprint patterns reflect the same long-lived RNAP holoenzyme transcription initiation intermediates observed with σ^70^. Though this study cannot definitively rule out that this observation can be attributed to limitations of formaldehyde crosslinking, several pieces of supporting information suggest otherwise. First, the same binding pattern observed in ChIP-exo studies performed on the native *crp* gene using an anti-Crp antibody were also observed in a c-*myc*-tagged *crp* gene fusion strain of *E*. *coli* K12 using an anti-c-myc antibody. Second, the closely related transcription factor, Fnr, yielded analogous ChIP-exo profiles to Crp on the template and nontemplate strands whereas the ChIP-exo profile of ArcA, a predominantly repressing transcription factor [48], showed a completely different binding profile. ArcA, as well as previously published work on Fur [35], demonstrate strong centering on the DNA motif and a narrower footprint compared with Crp and Fnr. Third, systematic mutations disrupting Crp/RNAP interactions revealed a significant loss in ChIP signal in Class I and Class II activating promoters but little disruption to the Class III promoters. Therefore, ChIP-exo binding sites associated with RNAP were eliminated in Crp-RNAP binding deficient mutants, whereas, binding events not associated with RNAP, namely those distant from the TSS, were still observed and motif centered. In fact, all conditions tested showed a subset of Crp binding sites that are motif centered. Thus, the alignment of ChIP-exo data relative to the TSS and the advancement of the template strand ChIP-exo distribution to the +20 position appear to be characteristic of transcriptional activation, whereas peak regions aligning relative to the motif sequence are not. Nevertheless, subsequent orthogonal confirmation of observations resulting from ChIP-exo studies would be beneficial though doing so under *in vivo* conditions and at the genome-scale is currently not feasible.

The vast majority of *in vitro* Crp studies have focused on the mechanism of recruitment and this transcription factor’s role in transcription initiation. However, a series of experiments were performed that deciphered the role of Crp in transcription after the RP_O_ complex was formed. A heparin challenge was applied to different Crp promoter classes to displace Crp from the open ternary complex [49,50]. In every promoter characterized, removal of Crp was inconsequential to transcriptional output upon open complex formation. These studies established that Crp plays a role in the recruitment of RNAP holoenzyme and also the isomerization of RNAP holoenzyme to form the open complex [1,2]. Thereafter, Crp’s presence or absence at the DNA binding site has no impact on transcription. Therefore, it is plausible that as RNAP holoenzyme transverses through the post-recruitment stages of transcription initiation, Crp is displaced from the DNA binding motif but remains bound to RNAP holoenzyme until promoter escape (S6 Fig). This hypothesis would explain the Crp ChIP-exo footprinting pattern that closely resembles that of RNAP holoenzyme and the poor protection of Class I and Class II DNA motif sequences. In addition to the data discussed above, Crp binding profiles in the presence of rifampicin indicate that Crp remains bound to the RNAP holoenzyme up to and including TEC formation (S4 Fig).

However, the data generated in this study alone cannot resolve what drives Crp/DNA dissociation to occur or how the release of Crp occurs from RNAP holoenzyme. The mechanisms driving σ factor release have proven to be elusive [21,51–53] and the release of transcriptional activators will likely be just as elusive. It is thought that the energy needed for promoter escape is established through a stressed intermediate resulting from scrunching [54,55]. This stressed intermediate may break the bonds between the σ factor and RNAP enabling RNAP to proceed to the elongation stage of transcription while the σ factor is retained at the promoter or dislodged from the promoter. Perhaps scrunching provides sufficient energy to also break the bonds formed between Crp, the σ factor, and RNAP, thereby enabling full transition into transcription elongation.

The detailed molecular interactions elucidated here reflect transitions of RNAP during transcription initiation at the genome-scale. This study is merely a starting point with numerous potential applications for ChIP-exo in studying promoter dynamics. The challenge will be to integrate multi-scale approaches such that we advance beyond studying just binary interactions of transcriptional regulators and begin to quantitatively unravel the molecular dynamics of transcription initiation. We believe that the datasets and analytical approaches utilized here provide a key component towards possibly reconstructing a quantitative, mechanistic, predictive model of promoter dynamics at the genome-scale.

## Experimental procedures

### Strains and culturing conditions

*Escherichia coli* MG1655 cells and derivatives thereof were used for all experiments. Fnr-8-myc, and ArcA-8-myc tagged strains were previously constructed [56]. The Δ*crp* strain was generated by replacing native gene with a kanamycin resistance marker from start codon to stop codon using the λ red mediated gene replacement method described [57]. The Δ*crp* was used as a basis for constructing the ΔAr1, ΔAr2 and ΔAr1ΔAr2 mutant strains using a modification of the λ red mediated gene replacement method. Briefly, plasmids carrying the different Ar mutant sequences were *de novo* synthesized using GeneArt (Life Technologies) with restriction sites at the 5’ and 3’ end of the gene. The gene was digested from GeneArt plasmids and ligated into the pKD3 plasmid directly upstream of the chloramphenicol (Cm) resistance gene. Resulting plasmids have the Ar mutant-*crp* gene, followed by the FRT flanked Cm resistance cassette as in pKD3 plasmid. Linear PCR products were amplified from resulting modified pKD3 plasmids using primers with 5’ overhangs with homology directly upstream of the start codon and downstream of the stop codon of *crp* gene to direct the insertion. This PCR product was transformed into electrocompetent Δ*crp E*. *coli* K12 carrying the pKD46 plasmid, and selected by Cm resistance, correct insertions were verified by Sanger sequencing. The Cm resistance gene was then removed from confirmed mutant strains by FLP recombinase excision transforming with pCP20 plasmid as previously described [57]. The ΔAr1 mutant introduces a mutation to the Ar1 region, HL159, previously determined to break contacts between Ar1 and the ɑ subunit of RNAP [13,58]. The ΔAr2 mutant does the same for Ar2 but introduces two mutations, KE101 and HY19 [58]. The ΔAr1ΔAr2 strain carries the HL159 mutation and the KE101 mutation.

M9 minimal media was used for all cultures with 2 g/L of glucose, fructose, or glycerol. For σ^70^, Crp, Δ*crp*, ΔAr1, ΔAr2, and ΔAr1ΔAr2 experiments, cultures were grown aerobically in shake flasks. Rifampicin conditions were incubated in the presence of rifampicin (50 μg/mL final concentration) for 20 min prior to crosslinking as previously described [59]. Fnr and ArcA experiments were conducted similarly but grown under anaerobic conditions.

### ChIP-exo experiments

The ChIP-exo protocol was adapted from Rhee and et al. for the Illumina platforms with the following modifications [33]. DNA crosslinking, fragmentation, and immunoprecipitation were performed as previously described [60] unless otherwise stated. Clarified lysate was continuously sonicated at 4 °C using a sonicator bell (6W) for 30 min. Antibodies used in this study are: anti-Crp (Neoclone N0004), anti-σ^70^ (Neoclone WP004), and anti-Myc (Santa Cruz Biotechnology sc-40). Immunocomplexes were captured using Pan Mouse IgG Dynabeads (Life Technologies). The following library preparation steps were sequentially performed while the protein/DNA/antibody complexes were bound to the magnetic beads: end repair (NEB End Repair Module), dA tailing (NEB dA-Tailing Module), adaptor 2 ligation (NEB Quick Ligase), nick repair (NEB PreCR Repair Mix), lambda exonuclease treatment (NEB), and RecJ_f_ exonuclease treatment (NEB). A series of step-down washes were done between all steps using buffers previously described [60]. Strand regeneration and library preparation followed the approach of Rhee et al. with the exception of a 3’ overhang removal step after the first adaptor ligation and prior to PCR enrichment by treating with T4 DNA Polymerase for 20 min at 12 °C. Libraries were sequenced on an Illumina MiSeq. Reads were aligned to the NC_000913.2 genome using bowtie2 [61] with default settings. Peak calling was performed using GPS in the GEMS analysis package [62] with the ChIP-exo default read distribution file with the following parameter settings: mrc 20, smooth 3, no read filtering, and no filter predicted events. GPS was used over GEMS because GEMS peak boundaries are influenced by motif identification whereas GPS is not. ChIP-peak calls were manually curated for anti-Crp (wt and Ar mutant strains) and anti-Myc (Fnr, and ArcA) for all substrates and conditions. A superset of GPS peak calls across all anti-Crp conditions was analyzed for presence/absence in each individual condition.

### Gene expression

Gene expression analysis was performed using a strand-specific, paired-end RNA-seq protocol using the dUTP method [63]. Total RNA was isolated and purified using the Qiagen Rneasy Kit with on-column DNase treatment. Total RNA was depleted of ribosomal RNAs using Epicentre’s RiboZero rRNA removal kit. rRNA depleted RNA was then primed using random hexamers and reverse transcribed using SuperScript III (Life Technologies). Sequencing was performed on an Illumina MiSeq. Reads were mapped to the NC_000913.2 reference genome using the default settings in bowtie2 [61]. Datasets were quantified using cuffdiff in the cufflinks package to generate FPKM (Framents Per Kilobase per Million reads mapped) values for all genes [64].

## Supporting information

S1 Fig σ^70^ ChIP-exo profile on fructose and glucose minimal media. (A) The mean 5’ tag density profile is shown for σ^70^ grown on fructose minimal media for the template (dashed black trace) and the nontemplate strand (solid black trace). Also shown are the peak center positions relative to the TSS (blue bars). (B) The mean 5’ tag density profile is shown for σ^70^ grown on glucose minimal media for the template (dashed black trace) and the nontemplate strand (solid black trace). Also shown are the peak center positions relative to the TSS (blue bars).
(EPS)

S2 Fig Crp ChIP-exo on activating and repressing substrates. (A) The mean 5’ tag density profile is shown for Crp grown on fructose minimal media (a Crp activating condition) for the template (dashed black trace) and the nontemplate strand (solid black trace). Also shown are the peak center positions (blue bars) and predicted Crp binding sites (gray bars) relative to the TSS. This profile closely resembles the Crp ChIP-exo profile generated on glycerol minimal media. (B) The mean 5’ tag density profile is shown for Crp grown on glucose minimal media (a Crp repressing condition) for the template (dashed black trace) and the nontemplate strand (solid black trace). Also shown are the peak center positions (blue bars) and predicted Crp binding sites (gray bars) relative to the TSS. The peak regions detected do not align well to the TSS, a stark divergence from profiles observed on activating carbon sources.
(EPS)

S3 Fig Correlation plots of ChIP-exo data generated on Δ*crp*. A whole genome correlation plot is shown for the Δ*crp* ChIP-exo profiles generated using the anti-crp antibody shows poor correlation between biological replicates.
(EPS)

S4 Fig Rifampicin treated Crp ChIP-exo profile. Crp protected footprints were examined by adding rif to the culture medium prior to harvest capture RNAP holoenzyme at stable intermediates prior to and including the ITC. Crp ChIP-exo distributions are shown for shared peak regions between rif-treated and wild type cultures grown on glycerol. The protected footprint regions mirror those found in the non-rif treated samples.
(EPS)

S5 Fig Comparison of ChIP-exo profiles for wild type, ΔAr1, ΔAr2, and ΔAr1ΔAr2 mutant strains. The mean 5’ tag density profiles are shown for wild type Crp, ΔAr1, ΔAr2, and ΔAr1ΔAr2 mutant strains from top to bottom. This plot shows the systematic los off TSS-centered peak regions with successive perturbation of RNAP holoenzyme/Crp interactions. Ultimately, the ΔAr1ΔAr2 profile does not resemble the profiles obtained under activating conditions that indicated protection of transcription initiation at post-recruitment stable intermediates.
(EPS)

S6 Fig Proposed model for Crp family binding interactions at Class I and Class II activating promoters. Shown is an illustration of Class I and Class II promoter models for Crp binding events. Initial recruitment involves a relatively short-lived complex consisting of the motif sequence(s), Crp, and RNAP holoenzyme. This complex observed using *in vitro* footprinting studies are not observed under physiological conditions studied performed using ChIP-exo. Instead, the longer-lived Crp/RNAP holoenzyme complex associated with post recruitment stable intermediates (RP_O_ shown) is observed leaving the motif sequence available for nuclease digestion.
(EPS)

S1 Table List of σ^70^ promoters classified by peak width. This table contains the identity of the 699 promoters which make up the trimodal distribution in Fig 1 of the manuscript. The first column is the transcription start site which all of the peak distributions shown in the paper were centered off of. The second column is genomic strand. The third column is the ID of the transcription unit. The third and fourth columns contain lists of the gene names and gene locus IDs contained within the transcription unit of the corresponding promoter. The fifth column contains the peak mode. Mode 1 corresponds to peaks that were 5-20bp in width, Mode 2 corresponds to peaks that were 21-40bp in width, Mode 3 corresponds to peaks that were 41-60bp in width.
(CSV)

S1 Text Supporting text. (DOCX)

## References

[1] Busby S, Ebright RH (1999) Transcription activation by catabolite activator protein (CAP). J Mol Biol 293: 199–213. doi: 10.1006/jmbi.1999.3161 10550204 10.1006/jmbi.1999.3161

[2] Lawson CL, Swigon D, Murakami KS, Darst SA, Berman HM, Ebright RH (2004) Catabolite activator protein: DNA binding and transcription activation. Curr Opin Struct Biol 14: 10–20. doi: 10.1016/j.sbi.2004.01.012 15102444 10.1016/j.sbi.2004.01.012 PMC2765107

[3] Kolb A, Busby S, Buc H, Garges S, Adhya S (1993) Transcriptional regulation by cAMP and its receptor protein. Annu Rev Biochem 62: 749–795. doi: 10.1146/annurev.bi.62.070193.003533 8394684 10.1146/annurev.bi.62.070193.003533

[4] Ebright RH, Cossart P, Gicquel-Sanzey B, Beckwith J (1984) Molecular basis of DNA sequence recognition by the catabolite gene activator protein: detailed inferences from three mutations that alter DNA sequence specificity. Proc Natl Acad Sci U S A 81: 7274–7278. 6390433 10.1073/pnas.81.23.7274 PMC392128

[5] de Crombrugghe B, Busby S, Buc H (1984) Cyclic AMP receptor protein: role in transcription activation. Science 224: 831–838. 6372090 10.1126/science.6372090

[6] Berg OG, von Hippel PH (1988) Selection of DNA binding sites by regulatory proteins. II. The binding specificity of cyclic AMP receptor protein to recognition sites. J Mol Biol 200: 709–723. 3045325 10.1016/0022-2836(88)90482-2

[7] Stormo GD, Hartzell GW 3rd (1989) Identifying protein-binding sites from unaligned DNA fragments. Proc Natl Acad Sci U S A 86: 1183–1187. 2919167 10.1073/pnas.86.4.1183 PMC286650

[8] Rhodius VA, Busby SJ (2000) Interactions between activating region 3 of the *Escherichia coli* cyclic AMP receptor protein and region 4 of the RNA polymerase sigma(70) subunit: application of suppression genetics. J Mol Biol 299: 311–324. doi: 10.1006/jmbi.2000.3737 10860740 10.1006/jmbi.2000.3737

[9] Lonetto MA, Rhodius V, Lamberg K, Kiley P, Busby S, Gross C (1998) Identification of a contact site for different transcription activators in region 4 of the *Escherichia coli* RNA polymerase sigma70 subunit. J Mol Biol 284: 1353–1365. doi: 10.1006/jmbi.1998.2268 9878355 10.1006/jmbi.1998.2268

[10] Savery NJ, Lloyd GS, Kainz M, Gaal T, Ross W, Ebright RH, et al (1998) Transcription activation at Class II CRP-dependent promoters: identification of determinants in the C-terminal domain of the RNA polymerase alpha subunit. EMBO J 17: 3439–3447. doi: 10.1093/emboj/17.12.3439 9628879 10.1093/emboj/17.12.3439 PMC1170680

[11] Zhou Y, Merkel TJ, Ebright RH (1994) Characterization of the activating region of *Escherichia coli* catabolite gene activator protein (CAP). II. Role at Class I and class II CAP-dependent promoters. J Mol Biol 243: 603–610. 7966285 10.1016/0022-2836(94)90035-3

[12] Niu W, Zhou Y, Dong Q, Ebright YW, Ebright RH (1994) Characterization of the activating region of *Escherichia coli* catabolite gene activator protein (CAP). I. Saturation and alanine-scanning mutagenesis. J Mol Biol 243: 595–602. 7966284 10.1016/0022-2836(94)90034-5

[13] West D, Williams R, Rhodius V, Bell A, Sharma N, Zou C (1993) Interactions between the *Escherichia coli* cyclic AMP receptor protein and RNA polymerase at class II promoters. Mol Microbiol 10: 789–797. 7934841 10.1111/j.1365-2958.1993.tb00949.x

[14] Zhou Y, Zhang X, Ebright RH (1993) Identification of the activating region of catabolite gene activator protein (CAP): isolation and characterization of mutants of CAP specifically defective in transcription activation. Proc Natl Acad Sci U S A 90: 6081–6085. 8392187 10.1073/pnas.90.13.6081 PMC46871

[15] Zhou Y, Busby S, Ebright RH (1993) Identification of the functional subunit of a dimeric transcription activator protein by use of oriented heterodimers. Cell 73: 375–379. 8477449 10.1016/0092-8674(93)90236-j

[16] Benoff B, Yang H, Lawson CL, Parkinson G, Liu J, Blatter E (2002) Structural basis of transcription activation: the CAP-alpha CTD-DNA complex. Science 297: 1562–1566. doi: 10.1126/science.1076376 12202833 10.1126/science.1076376

[17] Parkinson G, Gunasekera A, Vojtechovsky J, Zhang X, Kunkel TA, Berman H, et al (1996) Aromatic hydrogen bond in sequence-specific protein DNA recognition. Nat Struct Biol 3: 837–841. 8836098 10.1038/nsb1096-837

[18] Parkinson G, Wilson C, Gunasekera A, Ebright YW, Ebright RH, Berman HM (1996) Structure of the CAP-DNA complex at 2.5 angstroms resolution: a complete picture of the protein-DNA interface. J Mol Biol 260: 395–408. 8757802 10.1006/jmbi.1996.0409

[19] Schultz SC, Shields GC, Steitz TA (1991) Crystal structure of a CAP-DNA complex: the DNA is bent by 90 degrees. Science 253: 1001–1007. 1653449 10.1126/science.1653449

[20] Galas DJ, Schmitz A (1978) DNAse footprinting: a simple method for the detection of protein-DNA binding specificity. Nucleic Acids Res 5: 3157–3170. 212715 10.1093/nar/5.9.3157 PMC342238

[21] Hsu LM (2002) Promoter clearance and escape in prokaryotes. Biochim Biophys Acta 1577: 191–207. 12213652 10.1016/s0167-4781(02)00452-9

[22] Record M, Reznikoff WS, Craig ML, McQuade KL, Schlax PJ (1996) *Escherichia coli* RNA polymerase (Es70), promoters, and the kinetics of the steps of transcription initiation Escherichia coli and Salmonella Cellular and Molecular Biology Edited by Neidhardt FC et al ASM Press, Washington DC: 792–821.

[23] Saecker RM, Record MT Jr., Dehaseth PL (2011) Mechanism of bacterial transcription initiation: RNA polymerase—promoter binding, isomerization to initiation-competent open complexes, and initiation of RNA synthesis. J Mol Biol 412: 754–771. doi: 10.1016/j.jmb.2011.01.018 21371479 10.1016/j.jmb.2011.01.018 PMC3440003

[24] Hook-Barnard IG, Hinton DM (2007) Transcription initiation by mix and match elements: flexibility for polymerase binding to bacterial promoters. Gene Regul Syst Bio 1: 275–293. 19119427 PMC2613000

[25] Davis CA, Bingman CA, Landick R, Record MT Jr., Saecker RM (2007) Real-time footprinting of DNA in the first kinetically significant intermediate in open complex formation by *Escherichia coli* RNA polymerase. Proc Natl Acad Sci U S A 104: 7833–7838. doi: 10.1073/pnas.0609888104 17470797 10.1073/pnas.0609888104 PMC1876533

[26] Kovacic RT (1987) The 0 °C closed complexes between *Escherichia coli* RNA polymerase and two promoters, T7-A3 and *lac*UV5. J Biol Chem 262: 13654–13661. 3308880

[27] Schickor P, Metzger W, Werel W, Lederer H, Heumann H (1990) Topography of intermediates in transcription initiation of *E*.*coli*. EMBO J 9: 2215–2220. 2192861 10.1002/j.1460-2075.1990.tb07391.x PMC551945

[28] Sclavi B, Zaychikov E, Rogozina A, Walther F, Buckle M, Heumann H (2005) Real-time characterization of intermediates in the pathway to open complex formation by *Escherichia coli* RNA polymerase at the T7A1 promoter. Proc Natl Acad Sci U S A 102: 4706–4711. doi: 10.1073/pnas.0408218102 15738402 10.1073/pnas.0408218102 PMC555702

[29] Belyaeva TA, Rhodius VA, Webster CL, Busby SJ (1998) Transcription activation at promoters carrying tandem DNA sites for the *Escherichia coli* cyclic AMP receptor protein: organisation of the RNA polymerase alpha subunits. J Mol Biol 277: 789–804. doi: 10.1006/jmbi.1998.1666 9545373 10.1006/jmbi.1998.1666

[30] Belyaeva TA, Bown JA, Fujita N, Ishihama A, Busby SJ (1996) Location of the C-terminal domain of the RNA polymerase alpha subunit in different open complexes at the *Escherichia coli* galactose operon regulatory region. Nucleic Acids Res 24: 2242–2251. 8710492 10.1093/nar/24.12.2243 PMC145949

[31] Attey A, Belyaeva T, Savery N, Hoggett J, Fujita N, Ishihama A, et al (1994) Interactions between the cyclic AMP receptor protein and the alpha subunit of RNA polymerase at the *Escherichia coli* galactose operon P1 promoter. Nucleic Acids Res 22: 4375–4380. 7971267 10.1093/nar/22.21.4375 PMC308469

[32] Kolb A, Igarashi K, Ishihama A, Lavigne M, Buckle M, Buc H (1993) *E*. *coli* RNA polymerase, deleted in the C-terminal part of its alpha-subunit, interacts differently with the cAMP-CRP complex at the lacP1 and at the galP1 promoter. Nucleic Acids Res 21: 319–326. 8382795 10.1093/nar/21.2.319 PMC309109

[33] Rhee HS, Pugh BF (2011) Comprehensive genome-wide protein-DNA interactions detected at single-nucleotide resolution. Cell 147: 1408–1419. doi: 10.1016/j.cell.2011.11.013 22153082 10.1016/j.cell.2011.11.013 PMC3243364

[34] Rhee HS, Pugh BF (2012) Genome-wide structure and organization of eukaryotic pre-initiation complexes. Nature 483: 295–301. doi: 10.1038/nature10799 22258509 10.1038/nature10799 PMC3306527

[35] Seo SW, Kim D, Latif H, O’Brien EJ, Szubin R, Palsson BO (2014) Deciphering Fur transcriptional regulatory network highlights its complex role beyond iron metabolism in *Escherichia coli*. Nat Commun 5: 4910 doi: 10.1038/ncomms5910 25222563 10.1038/ncomms5910 PMC4167408

[36] Germer J, Becker G, Metzner M, Hengge-Aronis R (2001) Role of activator site position and a distal UP-element half-site for sigma factor selectivity at a CRP/H-NS-activated sigma(s)-dependent promoter in *Escherichia coli*. Mol Microbiol 41: 705–716. 11532138 10.1046/j.1365-2958.2001.02548.x

[37] Kristensen HH, Valentin-Hansen P, Sogaard-Andersen L (1997) Design of CytR regulated, cAMP-CRP dependent class II promoters in *Escherichia coli*: RNA polymerase-promoter interactions modulate the efficiency of CytR repression. J Mol Biol 266: 866–876. 9086266 10.1006/jmbi.1996.0852

[38] Pedersen H, Dall J, Dandanell G, Valentin-Hansen P (1995) Gene-regulatory modules in *Escherichia coli*: nucleoprotein complexes formed by cAMP-CRP and CytR at the *nupG* promoter. Mol Microbiol 17: 843–853. 8596434 10.1111/j.1365-2958.1995.mmi_17050843.x

[39] Williams RM, Rhodius VA, Bell AI, Kolb A, Busby SJ (1996) Orientation of functional activating regions in the *Escherichia coli* CRP protein during transcription activation at class II promoters. Nucleic Acids Res 24: 1112–1118. 8604346 10.1093/nar/24.6.1112 PMC145741

[40] Mymryk JS, Archer TK (1994) Detection of transcription factor binding *in vivo* using lambda exonuclease. Nucleic Acids Res 22: 4344–4345. 7937164 10.1093/nar/22.20.4344 PMC331963

[41] Valentin-Hansen P (1982) Tandem CRP binding sites in the deo operon of *Escherichia coli* K-12. EMBO J 1: 1049–1054. 6329724 10.1002/j.1460-2075.1982.tb01295.x PMC553161

[42] Campbell EA, Korzheva N, Mustaev A, Murakami K, Nair S, Goldfarb A, et al (2001) Structural mechanism for rifampicin inhibition of bacterial rna polymerase. Cell 104: 901–912. 11290327 10.1016/s0092-8674(01)00286-0

[43] Serandour AA, Brown GD, Cohen JD, Carroll JS (2013) Development of an Illumina-based ChIP-exonuclease method provides insight into FoxA1-DNA binding properties. Genome Biol 14: R147 doi: 10.1186/gb-2013-14-12-r147 24373287 10.1186/gb-2013-14-12-r147 PMC4053927

[44] Federowicz S, Kim D, Ebrahim A, Lerman J, Nagarajan H, Cho BK, et al (2014) Determining the control circuitry of redox metabolism at the genome-scale. PLoS Genet 10: e1004264 doi: 10.1371/journal.pgen.1004264 24699140 10.1371/journal.pgen.1004264 PMC3974632

[45] Reppas NB, Wade JT, Church GM, Struhl K (2006) The transition between transcriptional initiation and elongation in *E*. *coli* is highly variable and often rate limiting. Mol Cell 24: 747–757. doi: 10.1016/j.molcel.2006.10.030 17157257 10.1016/j.molcel.2006.10.030

[46] Wade JT, Struhl K (2004) Association of RNA polymerase with transcribed regions in *Escherichia coli*. Proc Natl Acad Sci U S A 101: 17777–17782. doi: 10.1073/pnas.0404305101 15596728 10.1073/pnas.0404305101 PMC539717

[47] Wade JT, Struhl K (2008) The transition from transcriptional initiation to elongation. Curr Opin Genet Dev 18: 130–136. doi: 10.1016/j.gde.2007.12.008 18282700 10.1016/j.gde.2007.12.008 PMC2563432

[48] Green J, Paget MS (2004) Bacterial redox sensors. Nat Rev Microbiol 2: 954–966. doi: 10.1038/nrmicro1022 15550941 10.1038/nrmicro1022

[49] Tagami H, Aiba H (1998) A common role of CRP in transcription activation: CRP acts transiently to stimulate events leading to open complex formation at a diverse set of promoters. EMBO J 17: 1759–1767. doi: 10.1093/emboj/17.6.1759 9501097 10.1093/emboj/17.6.1759 PMC1170523

[50] Tagami H, Aiba H (1995) Role of CRP in transcription activation at *Escherichia coli lac* promoter: CRP is dispensable after the formation of open complex. Nucleic Acids Res 23: 599–605. 7899079 10.1093/nar/23.4.599 PMC306726

[51] Bai L, Santangelo TJ, Wang MD (2006) Single-molecule analysis of RNA polymerase transcription. Annu Rev Biophys Biomol Struct 35: 343–360. doi: 10.1146/annurev.biophys.35.010406.150153 16689640 10.1146/annurev.biophys.35.010406.150153

[52] Herbert KM, Greenleaf WJ, Block SM (2008) Single-molecule studies of RNA polymerase: motoring along. Annu Rev Biochem 77: 149–176. doi: 10.1146/annurev.biochem.77.073106.100741 18410247 10.1146/annurev.biochem.77.073106.100741 PMC2854675

[53] Mooney RA, Darst SA, Landick R (2005) Sigma and RNA polymerase: an on-again, off-again relationship? Mol Cell 20: 335–345. doi: 10.1016/j.molcel.2005.10.015 16285916 10.1016/j.molcel.2005.10.015

[54] Kapanidis AN, Margeat E, Ho SO, Kortkhonjia E, Weiss S, Ebright RH (2006) Initial transcription by RNA polymerase proceeds through a DNA-scrunching mechanism. Science 314: 1144–1147. doi: 10.1126/science.1131399 17110578 10.1126/science.1131399 PMC2754788

[55] Revyakin A, Liu C, Ebright RH, Strick TR (2006) Abortive initiation and productive initiation by RNA polymerase involve DNA scrunching. Science 314: 1139–1143. doi: 10.1126/science.1131398 17110577 10.1126/science.1131398 PMC2754787

[56] Cho BK, Knight EM, Palsson BO (2006) PCR-based tandem epitope tagging system for *Escherichia coli* genome engineering. Biotechniques 40: 67–72. 16454042 10.2144/000112039

[57] Datsenko KA, Wanner BL (2000) One-step inactivation of chromosomal genes in *Escherichia coli* K-12 using PCR products. Proc Natl Acad Sci U S A 97: 6640–6645. doi: 10.1073/pnas.120163297 10829079 10.1073/pnas.120163297 PMC18686

[58] Rhodius VA, West DM, Webster CL, Busby SJ, Savery NJ (1997) Transcription activation at class II CRP-dependent promoters: the role of different activating regions. Nucleic Acids Res 25: 326–332. 9016561 10.1093/nar/25.2.326 PMC146447

[59] Cho BK, Zengler K, Qiu Y, Park YS, Knight EM, Barrett CL, et al (2009) The transcription unit architecture of the *Escherichia coli* genome. Nat Biotechnol 27: 1043–1049. doi: 10.1038/nbt.1582 19881496 10.1038/nbt.1582 PMC3832199

[60] Cho BK, Barrett CL, Knight EM, Park YS, Palsson BO (2008) Genome-scale reconstruction of the Lrp regulatory network in *Escherichia coli*. Proc Natl Acad Sci U S A 105: 19462–19467. doi: 10.1073/pnas.0807227105 19052235 10.1073/pnas.0807227105 PMC2614783

[61] Langmead B, Salzberg SL (2012) Fast gapped-read alignment with Bowtie 2. Nat Methods 9: 357–359. doi: 10.1038/nmeth.1923 22388286 10.1038/nmeth.1923 PMC3322381

[62] Guo Y, Mahony S, Gifford DK (2012) High resolution genome wide binding event finding and motif discovery reveals transcription factor spatial binding constraints. PLoS Comput Biol 8: e1002638 doi: 10.1371/journal.pcbi.1002638 22912568 10.1371/journal.pcbi.1002638 PMC3415389

[63] Levin JZ, Yassour M, Adiconis X, Nusbaum C, Thompson DA, Friedman N, et al (2010) Comprehensive comparative analysis of strand-specific RNA sequencing methods. Nat Methods 7: 709–715. doi: 10.1038/nmeth.1491 20711195 10.1038/nmeth.1491 PMC3005310

[64] Trapnell C, Hendrickson DG, Sauvageau M, Goff L, Rinn JL, Pachter L (2013) Differential analysis of gene regulation at transcript resolution with RNA-seq. Nat Biotechnol 31: 46–53. doi: 10.1038/nbt.2450 23222703 10.1038/nbt.2450 PMC3869392

