# Supplementary material for: ChIP-exo interrogation of Crp, DNA, and RNAP holoenzyme interactions
Source: PLoS One. 2018 May 17;13(5):e0197272. doi: 10.1371/journal.pone.0197272 (PMC5957442; doi:10.1371/journal.pone.0197272)
Supplement: S1 Text — (DOCX) [file pone.0197272.s008.docx]

**SUPPORTING INFORMATION**

**ChIP-exo interrogation of Crp, DNA, and RNAP holoenzyme interactions**

Haythem Latif^a,1,2^, Stephen Federowicz^a,1^, Ali Ebrahim^a^, Janna Tarasova^a^, Richard Szubin^a^,

Jose Utrilla^a^, Karsten Zengler^a^, Bernhard O. Palsson^a,b^

^a^Bioengineering Department, University of California San Diego

^b^Novo Nordisk Foundation Center for Biosustainability, Technical University of Denmark, 2800 Lyngby, Denmark

^1^Equally contributing authors

^2^To whom correspondence should be addressed: [halatif@ucsd.edu](mailto:Palsson@ucsd.edu) (H.L.)

Classification: BIOLOGICAL SCIENCES; Biophysics and Computational Biology

Keywords: Crp, ChIP-exo, RNA Polymerase, transcription initiation

**SUPPORTING TEXT**

**Resolution of ChIP-exo data localizes the TSS to within basepairs**

ChIP-chip and ChIP-seq data are enriched at bacterial promoters when the σ factor is immunoprecipitated [[1-8](#_ENREF_1)]. Ecocyc annotated TSSs were checked for σ^70^ ChIP-exo enrichment within ±200 bp for data generated on three different substrates—glucose, fructose, and glycerol (Fig 1A and S1 Fig). Like its predecessors, ChIP-exo peaks are consistently found near promoters. However, only the single-nucleotide resolution of ChIP-exo provides the exact genomic location bound by RNAP holoenzyme. Surprisingly, ChIP-exo data generated on σ^70^ is found to be a better proxy for the TSS than it is for the -35 and -10 promoter recognition sequence elements. The median position of the σ^70^ peak center is 5 bp downstream of the TSS for all three carbon sources (Fig 1A and S1 Fig). The spatial consistency of the σ^70^ peak center demonstrates the utility of ChIP-exo to approximate TSSs to within base pairs from where they exist and provides an orthogonal method to complement 5’ RACE-based TSS detection.

**Confirmation of Crp ChIP-exo binding sites with published data.**

Compared with published studies we are able to have a 91% (21/23) overlap with experimentally validated Crp binding sites [[42](#_ENREF_42)] and a 79% (23/29) overlap with previous ChIP-chip measurements that occurred in characterized Crp binding sites [[43](#_ENREF_43)]. We further see a 65% overlap (317/486) with all reported Crp target genes in RegulonDB [[44](#_ENREF_44)]. If one excludes the 65 genes conservatively estimated to be active only upon substrate specific inductions, this overlap increases to ~75% overlap (317/421) with Crp regulated genes found in RegulonDB.

**Brief review of Crp Activating regions**

The activating properties of Crp and many transcription factors is through stabilizing interactions with RNAP holoenzyme at the promoter [[45](#_ENREF_45), [46](#_ENREF_46)]. Molecular characterization studies and mutational analysis of Crp has revealed three activating regions (Ar’s) that make protein/protein interactions at specific positions in RNAP holoenzyme [[47](#_ENREF_47), [48](#_ENREF_48)]. The first, Ar1, interacts with either of the α subunit at the C-terminus and the HL159 mutation to Crp prevents this interaction from forming [[49](#_ENREF_49), [50](#_ENREF_50)]. This region is involved with activation at Class I and Class II promoters [[47](#_ENREF_47), [48](#_ENREF_48)]. The second region, Ar2, is only associated with Class II promoters and binds to the N-terminus of the α subunit. This bond was shown to be severely disrupted by introduction of two mutations to Crp, KE101 and HY19 [[49](#_ENREF_49)]. Lastly, a weaker interaction was found to occur at Ar3 between Crp and the σ factor [[51](#_ENREF_51), [52](#_ENREF_52)].

**REFERENCES**

1. Cho BK, Kim D, Knight EM, Zengler K, & Palsson BO (2014) Genome-scale reconstruction of the sigma factor network in *Escherichia coli*: topology and functional states. *BMC biology* 12:4.

2. Cho BK, Knight EM, & Palsson BO (2008) Genomewide identification of protein binding locations using chromatin immunoprecipitation coupled with microarray. *Methods in molecular biology* 439:131-145.

3. Cho BK*, et al.* (2009) The transcription unit architecture of the *Escherichia coli* genome. *Nature biotechnology* 27(11):1043-1049.

4. Herring CD*, et al.* (2005) Immobilization of *Escherichia coli* RNA polymerase and location of binding sites by use of chromatin immunoprecipitation and microarrays. *Journal of bacteriology* 187(17):6166-6174.

5. Kroger C*, et al.* (2012) The transcriptional landscape and small RNAs of *Salmonella enterica* serovar Typhimurium. *Proceedings of the National Academy of Sciences of the United States of America* 109(20):E1277-1286.

6. Qiu Y*, et al.* (2010) Structural and operational complexity of the *Geobacter sulfurreducens* genome. *Genome research* 20(9):1304-1311.

7. Reppas NB, Wade JT, Church GM, & Struhl K (2006) The transition between transcriptional initiation and elongation in *E. coli* is highly variable and often rate limiting. *Molecular cell* 24(5):747-757.

8. Wade JT, Struhl K, Busby SJ, & Grainger DC (2007) Genomic analysis of protein-DNA interactions in bacteria: insights into transcription and chromosome organization. *Molecular microbiology* 65(1):21-26.

9. Hook-Barnard IG & Hinton DM (2007) Transcription initiation by mix and match elements: flexibility for polymerase binding to bacterial promoters. *Gene regulation and systems biology* 1:275-293.

10. Hsu LM (2002) Promoter clearance and escape in prokaryotes. *Biochimica et biophysica acta* 1577(2):191-207.

11. Record M, Reznikoff WS, Craig ML, McQuade KL, & Schlax PJ (1996) *Escherichia coli* RNA polymerase (Es70), promoters, and the kinetics of the steps of transcription initiation. *Escherichia coli and Salmonella Cellular and Molecular Biology. Edited by Neidhardt FC et al. ASM Press, Washington DC*:792-821.

12. Saecker RM, Record MT, Jr., & Dehaseth PL (2011) Mechanism of bacterial transcription initiation: RNA polymerase - promoter binding, isomerization to initiation-competent open complexes, and initiation of RNA synthesis. *Journal of molecular biology* 412(5):754-771.

13. Rogozina A, Zaychikov E, Buckle M, Heumann H, & Sclavi B (2009) DNA melting by RNA polymerase at the T7A1 promoter precedes the rate-limiting step at 37 degrees C and results in the accumulation of an off-pathway intermediate. *Nucleic acids research* 37(16):5390-5404.

14. Schickor P, Metzger W, Werel W, Lederer H, & Heumann H (1990) Topography of intermediates in transcription initiation of *E.coli*. *The EMBO journal* 9(7):2215-2220.

15. Sclavi B*, et al.* (2005) Real-time characterization of intermediates in the pathway to open complex formation by *Escherichia coli* RNA polymerase at the T7A1 promoter. *Proceedings of the National Academy of Sciences of the United States of America* 102(13):4706-4711.

16. Bartlett MS, Gaal T, Ross W, & Gourse RL (1998) RNA polymerase mutants that destabilize RNA polymerase-promoter complexes alter NTP-sensing by *rrn* P1 promoters. *Journal of molecular biology* 279(2):331-345.

17. Kovacic RT (1987) The 0 °C closed complexes between *Escherichia coli* RNA polymerase and two promoters, T7-A3 and *lac*UV5. *The Journal of biological chemistry* 262(28):13654-13661.

18. Cook VM & Dehaseth PL (2007) Strand opening-deficient *Escherichia coli* RNA polymerase facilitates investigation of closed complexes with promoter DNA: effects of DNA sequence and temperature. *The Journal of biological chemistry* 282(29):21319-21326.

19. Davis CA, Bingman CA, Landick R, Record MT, Jr., & Saecker RM (2007) Real-time footprinting of DNA in the first kinetically significant intermediate in open complex formation by *Escherichia coli* RNA polymerase. *Proceedings of the National Academy of Sciences of the United States of America* 104(19):7833-7838.

20. Borukhov S, Sagitov V, Josaitis CA, Gourse RL, & Goldfarb A (1993) Two modes of transcription initiation *in vitro* at the *rrnB* P1 promoter of *Escherichia coli*. *The Journal of biological chemistry* 268(31):23477-23482.

21. Gourse RL (1988) Visualization and quantitative analysis of complex formation between *E. coli* RNA polymerase and an rRNA promoter *in vitro*. *Nucleic acids research* 16(20):9789-9809.

22. Newlands JT, Ross W, Gosink KK, & Gourse RL (1991) Factor-independent activation of *Escherichia coli* rRNA transcription. II. characterization of complexes of *rrnB* P1 promoters containing or lacking the upstream activator region with *Escherichia coli* RNA polymerase. *Journal of molecular biology* 220(3):569-583.

23. Craig ML*, et al.* (1998) DNA footprints of the two kinetically significant intermediates in formation of an RNA polymerase-promoter open complex: evidence that interactions with start site and downstream DNA induce sequential conformational changes in polymerase and DNA. *Journal of molecular biology* 283(4):741-756.

24. Spassky A, Kirkegaard K, & Buc H (1985) Changes in the DNA structure of the *lac* UV5 promoter during formation of an open complex with *Escherichia coli* RNA polymerase. *Biochemistry* 24(11):2723-2731.

25. Krummel B & Chamberlin MJ (1989) RNA chain initiation by *Escherichia coli* RNA polymerase. Structural transitions of the enzyme in early ternary complexes. *Biochemistry* 28(19):7829-7842.

26. Krummel B & Chamberlin MJ (1992) Structural analysis of ternary complexes of *Escherichia coli* RNA polymerase. Deoxyribonuclease I footprinting of defined complexes. *Journal of molecular biology* 225(2):239-250.

27. Straney DC & Crothers DM (1987) A stressed intermediate in the formation of stably initiated RNA chains at the *Escherichia coli lac* UV5 promoter. *Journal of molecular biology* 193(2):267-278.

28. Metzger W, Schickor P, & Heumann H (1989) A cinematographic view of *Escherichia coli* RNA polymerase translocation. *The EMBO journal* 8(9):2745-2754.

29. Zhilina E, Esyunina D, Brodolin K, & Kulbachinskiy A (2012) Structural transitions in the transcription elongation complexes of bacterial RNA polymerase during sigma-dependent pausing. *Nucleic acids research* 40(7):3078-3091.

30. Carpousis AJ & Gralla JD (1985) Interaction of RNA polymerase with *lac*UV5 promoter DNA during mRNA initiation and elongation. Footprinting, methylation, and rifampicin-sensitivity changes accompanying transcription initiation. *Journal of molecular biology* 183(2):165-177.

31. Wade JT & Struhl K (2004) Association of RNA polymerase with transcribed regions in *Escherichia coli*. *Proceedings of the National Academy of Sciences of the United States of America* 101(51):17777-17782.

32. Wade JT & Struhl K (2008) The transition from transcriptional initiation to elongation. *Current opinion in genetics & development* 18(2):130-136.

33. Gries TJ, Kontur WS, Capp MW, Saecker RM, & Record MT, Jr. (2010) One-step DNA melting in the RNA polymerase cleft opens the initiation bubble to form an unstable open complex. *Proceedings of the National Academy of Sciences of the United States of America* 107(23):10418-10423.

34. Saecker RM*, et al.* (2002) Kinetic studies and structural models of the association of *E. coli* σ70 RNA polymerase with the lambdaP(R) promoter: large scale conformational changes in forming the kinetically significant intermediates. *Journal of molecular biology* 319(3):649-671.

35. Tsodikov OV, Craig ML, Saecker RM, & Record MT, Jr. (1998) Quantitative analysis of multiple-hit footprinting studies to characterize DNA conformational changes in protein-DNA complexes: application to DNA opening by Esigma70 RNA polymerase. *Journal of molecular biology* 283(4):757-769.

36. Ring BZ, Yarnell WS, & Roberts JW (1996) Function of *E. coli* RNA polymerase σ factor σ70 in promoter-proximal pausing. *Cell* 86(3):485-493.

37. Brodolin K, Zenkin N, Mustaev A, Mamaeva D, & Heumann H (2004) The σ70 subunit of RNA polymerase induces *lac*UV5 promoter-proximal pausing of transcription. *Nature structural & molecular biology* 11(6):551-557.

38. Nickels BE, Mukhopadhyay J, Garrity SJ, Ebright RH, & Hochschild A (2004) The σ70 subunit of RNA polymerase mediates a promoter-proximal pause at the *lac* promoter. *Nature structural & molecular biology* 11(6):544-550.

39. Deighan P, Pukhrambam C, Nickels BE, & Hochschild A (2011) Initial transcribed region sequences influence the composition and functional properties of the bacterial elongation complex. *Genes & development* 25(1):77-88.

40. Hatoum A & Roberts J (2008) Prevalence of RNA polymerase stalling at *Escherichia coli* promoters after open complex formation. *Molecular microbiology* 68(1):17-28.

41. Tang GQ, Roy R, Bandwar RP, Ha T, & Patel SS (2009) Real-time observation of the transition from transcription initiation to elongation of the RNA polymerase. *Proceedings of the National Academy of Sciences of the United States of America* 106(52):22175-22180.

42. Zheng D, Constantinidou C, Hobman JL, & Minchin SD (2004) Identification of the Crp regulon using *in vitro* and *in vivo* transcriptional profiling. *Nucleic acids research* 32(19):5874-5893.

43. Grainger DC, Hurd D, Harrison M, Holdstock J, & Busby SJ (2005) Studies of the distribution of *Escherichia coli* cAMP-receptor protein and RNA polymerase along the *E. coli* chromosome. *Proceedings of the National Academy of Sciences of the United States of America* 102(49):17693-17698.

44. Salgado H*, et al.* (2013) RegulonDB v8.0: omics data sets, evolutionary conservation, regulatory phrases, cross-validated gold standards and more. *Nucleic acids research* 41(Database issue):D203-213.

45. Browning DF & Busby SJ (2004) The regulation of bacterial transcription initiation. *Nature reviews. Microbiology* 2(1):57-65.

46. Lee DJ, Minchin SD, & Busby SJ (2012) Activating transcription in bacteria. *Annual review of microbiology* 66:125-152.

47. Busby S & Ebright RH (1999) Transcription activation by catabolite activator protein (CAP). *Journal of molecular biology* 293(2):199-213.

48. Lawson CL*, et al.* (2004) Catabolite activator protein: DNA binding and transcription activation. *Current opinion in structural biology* 14(1):10-20.

49. Rhodius VA, West DM, Webster CL, Busby SJ, & Savery NJ (1997) Transcription activation at class II CRP-dependent promoters: the role of different activating regions. *Nucleic acids research* 25(2):326-332.

50. West D*, et al.* (1993) Interactions between the *Escherichia coli* cyclic AMP receptor protein and RNA polymerase at class II promoters. *Molecular microbiology* 10(4):789-797.

51. Rhodius VA & Busby SJ (2000) Transcription activation by the *Escherichia coli* cyclic AMP receptor protein: determinants within activating region 3. *Journal of molecular biology* 299(2):295-310.

52. Niu W, Kim Y, Tau G, Heyduk T, & Ebright RH (1996) Transcription activation at class II CAP-dependent promoters: two interactions between CAP and RNA polymerase. *Cell* 87(6):1123-1134.

**SUPPORTING TABLES**

**S1 Table. List of σ^70^ promoters classified by peak width**

This table contains the identity of the 699 promoters which make up the trimodal distribution in Figure 1 of the manuscript. The first column is the transcription start site which all of the peak distributions shown in the paper were centered off of. The second column is genomic strand. The third column is the ID of the transcription unit. The third and fourth columns contain lists of the gene names and gene locus IDs contained within the transcription unit of the corresponding promoter. The fifth column contains the peak mode. Mode 1 corresponds to peaks that were 5-20bp in width, Mode 2 corresponds to peaks that were 21-40bp in width, Mode 3 corresponds to peaks that were 41-60bp in width.

**SUPPORTING FIGURES**

**S1 Fig. σ^70^ ChIP-exo profile on fructose and glucose minimal media.**

1. The mean 5’ tag density profile is shown for σ^70^ grown on fructose minimal media for the template (dashed black trace) and the nontemplate strand (solid black trace). Also shown are the peak center positions relative to the TSS (blue bars).
2. The mean 5’ tag density profile is shown for σ^70^ grown on glucose minimal media for the template (dashed black trace) and the nontemplate strand (solid black trace). Also shown are the peak center positions relative to the TSS (blue bars).

**S2 Fig. Crp ChIP-exo on activating and repressing substrates.**

1. The mean 5’ tag density profile is shown for Crp grown on fructose minimal media (a Crp activating condition) for the template (dashed black trace) and the nontemplate strand (solid black trace). Also shown are the peak center positions (blue bars) and predicted Crp binding sites (gray bars) relative to the TSS. This profile closely resembles the Crp ChIP-exo profile generated on glycerol minimal media.
2. The mean 5’ tag density profile is shown for Crp grown on glucose minimal media (a Crp repressing condition) for the template (dashed black trace) and the nontemplate strand (solid black trace). Also shown are the peak center positions (blue bars) and predicted Crp binding sites (gray bars) relative to the TSS. The peak regions detected do not align well to the TSS, a stark divergence from profiles observed on activating carbon sources.

**S3 Fig. Correlation plots of ChIP-exo data generated on Δ*crp*.**

A whole genome correlation plot is shown for the Δ*crp* ChIP-exo profiles generated using the anti-crp antibody shows poor correlation between biological replicates.

**S4 Fig. Rifampicin treated Crp ChIP-exo profile.**

Crp protected footprints were examined by adding rif to the culture medium prior to harvest capture RNAP holoenzyme at stable intermediates prior to and including the ITC. Crp ChIP-exo distributions are shown for shared peak regions between rif-treated and wild type cultures grown on glycerol. The protected footprint regions mirror those found in the non-rif treated samples.

**S5 Fig. Comparison of ChIP-exo profiles for wild type, ΔAr1, ΔAr2, and ΔAr1ΔAr2 mutant strains.**

The mean 5’ tag density profiles are shown for wild type Crp, ΔAr1, ΔAr2, and ΔAr1ΔAr2 mutant strains from top to bottom. This plot shows the systematic los off TSS-centered peak regions with successive perturbation of RNAP holoenzyme/Crp interactions. Ultimately, the ΔAr1ΔAr2 profile does not resemble the profiles obtained under activating conditions that indicated protection of transcription initiation at post-recruitment stable intermediates.

**S6 Fig. Proposed model for Crp family binding interactions at Class I and Class II activating promoters.**

Shown is an illustration of Class I and Class II promoter models for Crp binding events. Initial recruitment involves a relatively short-lived complex consisting of the motif sequence(s), Crp, and RNAP holoenzyme. This complex observed using *in vitro* footprinting studies are not observed under physiological conditions studied performed using ChIP-exo. Instead, the longer-lived Crp/RNAP holoenzyme complex associated with post recruitment stable intermediates (RP_O_ shown) is observed leaving the motif sequence available for nuclease digestion.
